# Precise Genome Editing in Poultry and Its Application to Industries

**DOI:** 10.3390/genes11101182

**Published:** 2020-10-12

**Authors:** Jin Se Park, Kyung Youn Lee, Jae Yong Han

**Affiliations:** Department of Agricultural Biotechnology and Research Institute of Agriculture and Life Sciences, Seoul National University, Seoul 08826, Korea; bulgari77@snu.ac.kr (J.S.P.); leektmn@naver.com (K.Y.L.)

**Keywords:** poultry, PGCs, viral vector systems, CRISPR/Cas9, genome editing

## Abstract

Poultry such as chickens are valuable model animals not only in the food industry, but also in developmental biology and biomedicine. Recently, precise genome-editing technologies mediated by the CRISPR/Cas9 system have developed rapidly, enabling the production of genome-edited poultry models with novel traits that are applicable to basic sciences, agriculture, and biomedical industry. In particular, these techniques have been combined with cultured primordial germ cells (PGCs) and viral vector systems to generate a valuable genome-edited avian model for a variety of purposes. Here, we summarize recent progress in CRISPR/Cas9-based genome-editing technology and its applications to avian species. In addition, we describe further applications of genome-edited poultry in various industries.

## 1. Introduction

Poultry are a major source of protein in the form of meat and eggs. As the global human population grows, demand for livestock products will increase, and poultry will become an even more important source of protein [1]. However, climate change and outbreaks of viral diseases threaten poultry farming and the food supply: heat stress caused by climate change decreases feed intake and resistance to infection [2], and RNA viruses such as the avian influenza virus kill huge numbers of poultry, leading to enormous economic losses. Additionally, RNA viruses have the potential to evolve into zoonotic pathogens that directly threaten human health [3]. To address these environmental changes, new breeding strategies are required that can introduce desirable traits and allow livestock to endure potential threats.

Recently, genome-editing technologies such as the CRISPR/Cas9 system have developed rapidly, and these methods have been applied to the production of a variety of genome-edited animals, including livestock [4]. Genome editing can precisely modify DNA sequences of targeted genes to improve the productivity or disease resistance of livestock without introducing any undesired abnormalities. Since traditional selective breeding relies on naturally occurring genetic variations, it takes a long time to gain desirable traits. By contrast, using genome-editing technology, it is possible to introduce desirable genetic variations quickly, and as with selective breeding, no foreign sequence remains in the genome. Thus, genome editing represents an effective and safe method that can easily be combined with traditional breeding. Several studies have used genome editing to improve the productivity or disease resistance of livestock, including poultry [5,6,7].

Chicken eggs can also be used as an efficient platform for production of protein pharmaceuticals. An individual hen lays 300 eggs per year, and the composition of chicken egg white protein is relatively simple, making it easier to purify target proteins from eggs. Moreover, chicken egg white proteins have a characteristic *N*-glycosylation pattern that can improve the efficacy of protein drugs. Based on these properties, several studies have reported production and purification of protein drugs from egg white and demonstrated their efficacy [8,9,10,11,12]. Recently, an enzyme used to treat Wolman disease was purified from egg white and commercialized [13]. Additionally, egg white protein composition can be changed through genome editing, and foreign proteins can be produced at high levels by targeting the endogenous egg white protein locus [14,15]. Thus, editing can efficiently modulate egg white protein composition, and research on this topic will continue actively in the future.

We will begin by briefly introducing current genome-editing technologies based on the CRISPR/Cas9 system and their application to poultry. We will then discuss further applications of genome-edited poultry in industry.

## 2. Programmable Genome Editing Technologies Based on CRISPR/Cas9

The earliest programmable genome-editing tools were zinc finger nucleases (ZFNs), followed by transcription activator-like effector nucleases (TALENs) and later by CRISPR/Cas9 technology. With genome-editing tools, researchers can easily induce a double-strand break (DSB) at a target site, thereby knocking out a target gene or knocking in exogenous gene cassettes through delivery of a donor DNA template. In addition, base-editing and prime-editing technologies enable more efficient and precise modification of the genome without requiring a donor plasmid.

### 2.1. CRISPR-Cas9 Systems for Gene Knock-Out and Knock-In

The CRISPR/Cas9 system consists of a DSB-inducing endonuclease, Cas9, and a short single guide RNA (sgRNA) complementary to a specific region of the genome sequence [16]. When the CRISPR/Cas9 complex is delivered in vitro or in vivo, it induces a DSB in the target region; this break is preferentially repaired through error-prone nonhomologous end-joining (NHEJ), which induces small insertion or deletion mutations (indels), or through homology-directed repair (HDR) in the presence of a donor DNA template.

In general, NHEJ can be used to induce gene knockout by introducing indels into targeted genes, resulting in missense, nonsense, or frameshift mutations. NHEJ can also induce megabase-size deletions by delivering two sgRNAs targeting distant sites within the same gene [17,18].

HDR, a cellular repair mechanism performed by the homologous recombination (HR) pathway, can precisely modify the target genome when an exogenous donor template is present at the target site. However, spontaneous HR efficiency following delivery of the donor template is very inefficient, with a rate ranging from one event per 10^3^ to 10^9^ cells depending on cell type and cell state [19]. Homology-driven DNA recombination is stimulated when a DSB is induced by a rare-cutting endonuclease [20]. Since DSB can be induced by CRISPR/Cas9, genome modification is more efficient than with spontaneous HR; accordingly, HDR is widely used to introduce precise genome modifications such as point mutations or to insert exogenous gene cassettes for gene tracing. However, when a DSB is induced, it is preferentially repaired through NHEJ. Hence, to increase the efficiency of HDR-mediated repair, the target cells can be treated with SCR7, a chemical that interferes with NHEJ [21]; alternatively, Cas9 can be fused to CtIP, which promotes initiation of HDR [22]. Furthermore, an HDR-dependent knock-in strategy called CORRECT (‘Consecutive Re-guide or Re-Cas steps to Erase CRISPR/Cas-blocked Targets’) has been developed for scarless genome editing [23]. Based on these advances, CRISPR/Cas9 technology makes genome modification easier than ever and serves as a powerful and versatile tool. A great deal of progress has been made in CRISPR technology, and several Cas variants have been developed to overcome limitations on a targeting range, specifically to reach sequences that are inaccessible due to the lack of a nearby PAM motif [24,25]. In addition, variants such as spCas9-HF1 and eSpCas9 were developed by protein engineering to improve the targeting specificity of Cas9 [26,27]. However, more advanced technologies are still required to overcome limitations related to unintended indels and low HDR efficiency.

### 2.2. Base Editing Technologies

Recently, CRISPR/Cas9-mediated base editing technology was developed to achieve more precise genome modification. The cytosine base editor (CBE) consists of modified Cas9 (nickase Cas9 or dead Cas9), cytosine deaminase, sgRNA, and uracil *N*-glycosylase inhibitor (UGI), which can convert C to T (or G to A) without inducing a DSB [28,29]. Meanwhile, adenine base editor (ABE), another base editing technology, consists of nickase Cas9, mutated *E. coli* tRNA adenosine deaminase (TadA*), and sgRNA; this system can convert A to G (or T to C) by deaminating adenosine in DNA [30]. DNA base editing still induces indel mutations but at very low frequencies. Consequently, base editing yields more precise genome-editing outcomes than conventional CRISPR/Cas9 technology, and off-target effects are also relatively rare. Moreover, this system does not require exogenous donor template DNA. In light of these advantages, base-editing technology is widely used not only in the agricultural industry and in basic scientific research, but also for therapeutic purposes [31,32,33,34].

In addition to base editing in DNA, it is also possible to edit RNA. The RNA base editor (RBE) makes it possible to replace adenosine with inosine in RNA [35]. In contrast to DNA base editors, RBE consists of catalytically dead Cas13b, a type VI CRISPR/Cas system that binds to adenosine deaminase acting on RNA (ADAR); this system does not alter the genomic sequence because only the transcript is targeted. REPAIR (‘RNA Editing for Programmable A-to-I Replacement’) offers many advantages over DNA base editing. First, unlike Cas9, Cas13 does not require a PAM sequence and can theoretically edit any adenosine in the RNA sequence. Second, through the activity of the deaminase, adenosine is converted directly to inosine; thus the editing outcome is not affected by the endogenous cellular repair pathway, and editing can be performed efficiently even in post-mitotic cells. Using this system, disease-relevant mutations such as AVPR2 G878A in X-linked nephrogenic diabetes insipidus and *FANCC* G1517A in Fanconi anemia can be corrected in 23–35% of HEK293FT cells. Furthermore, protein engineering of ADAR has decreased proximal off-target editing and improved specificity (i.e., on-target efficiency) [35]. A more recently developed approach is LEAPER (‘Leveraging Endogenous ADAR for Programmable Editing of RNA’), which utilizes short engineered ADAR-recruiting RNAs (arRNAs) to recruit ADAR proteins to convert adenosine to inosine in RNA sequences [36]. This system has high editing efficiency, does not induce an immune response in the target cells, and is applicable to several kinds of primary cells, making it suitable for therapeutic use.

With the development of various base editors, precise modifications such as point mutations and single-nucleotide polymorphisms (SNPs) can be efficiently introduced, and the frequency of unintended indels and off-target effects has also been reduced [37].

### 2.3. Prime Editing Technologies

Recently developed base-editing technologies can efficiently perform four kinds of nucleotide-to-nucleotide conversions (C to T, G to A, A to G, and T to C). However, these technologies are limited in their ability to perform all 12 types of conversions, and precise modification of insertion and deletion mutations (indel) is difficult without introduction of a DSB or donor template. An innovative prime-editing technology consisting of nickase Cas9 (H840A), prime-editing extended guide RNA (pegRNA), and a mutated Moloney murine leukemia virus reverse transcriptase (M-MLV RT) overcomes these limitations and expands the ability of genome-editing technologies to achieve accurate genome modification [38]. The mutations in the M-MLV RT component improve processivity, thermostability, and binding affinity between the DNA and RNA substrates, thereby improving prime-editing efficiency. Moreover, in addition to the pegRNA, the use of another sgRNA that induces a nick on the non-edited strand can further increase editing efficiency. This form of prime-editing technology is the most advanced genome-editing technology developed to date, and has the ability to achieve precise genome modification with fewer off-target effects than conventional genome-editing technology. Despite the advantages of prime-editing technology, the protein structure is currently too large for efficient delivery in vitro or in vivo, and it cannot be used to induce precise modifications in large indel mutations. Therefore, for this approach to be applied more broadly, further research is needed to develop more optimized systems that can overcome current limitations and increase efficiency and specificity [39,40].

## 3. CRISPR-Cas9 Mediated Genome Editing in Poultry

Recently, the CRISPR/Cas9-mediated technologies described above have been used to generate various genome-edited poultry. Due to the physiological characteristics of poultry, pronuclear injection for genome editing cannot be conducted in these species [41]. Hence, genome-editing in poultry, especially in chicken, is performed using cultured primordial germ cells (PGCs). Recently, adenovirus-mediated genome editing was performed in quail, a species in which PGCs cannot be cultured in vitro. In addition, direct injection of a plasmid that encodes Cas9 and sgRNA into embryonic blood vessels achieved successful editing of germline cells and yielded genome-edited progeny. Here, we described the diverse genome-editing methods applied to poultry and recent progress in poultry genome editing.

### 3.1. PGCs Mediated Genome Editing in Poultry

In chicken, PGCs reside in the central region of the area pellucida at Eyal-Giladi and Kochav (EGK) stage X and migrate to the germinal crescent after primitive streak formation [42,43,44,45,46]. Subsequently, PGCs circulate in embryonic blood vessels and settle in the embryonic gonads [47]. Due to the unique migratory pathway of chicken PGCs, the cells can be isolated from various embryonic stages, and can also be cultured without losing germline potency. When these cultured PGCs are injected into recipient embryo blood vessels, they settle in the gonads, resulting in the production of a germline chimera [48,49,50,51]. A germline chimera produced by injection of genome-edited PGCs can produce genome-edited offspring. Recently, using cultured PGCs and CRISPR/Cas9 system, genome editing in chicken was successfully performed for several purposes.

The first genome-edited chicken produced by the CRISPR/Cas9 system was reported in 2016. In this study, ovomucoid (*OVM*), a major egg white allergen, was knocked out in chicken using CRISPR/Cas9 [15]. That study demonstrated that targeted mutagenesis mediated by the CRISPR/Cas9 system could be successfully performed in chicken PGCs, resulting in efficient production of genome-edited chickens. Subsequently, knockout of myostatin (*MSTN*) and G0/G1 switch gene 2 (*G0S2*) in chicken was performed using the CRISPR/Cas9 system [52,53].

Introduction of exogenous gene cassettes by HDR was successfully conducted in chicken using the CRISPR/Cas9 system. In 2016, a *loxP* site was introduced in the variable region (V region) of immunoglobulin heavy chain (IgH) using CRISPR/Cas9-mediated HDR, and a genome-edited chicken was produced [54]. This study was the first to show that HDR-mediated gene targeting can be successfully performed in chicken PGCs using CRISPR/Cas9. Subsequently, in 2018, human interferon β (hIFN-β) was targeted to the ovalbumin locus by CRISPR/Cas9-mediated HDR, and these chickens accumulated high levels of hIFN-β in egg white (1.86–4.42 mg/mL) [14]. Additionally, introduction of a point mutation that precisely deleted tryptophan residue 38 (W38) of chicken Na^+^/H^+^ exchanger type 1 (*chNHE1*) was successfully achieved by HDR [7].

The drawbacks of HDR are its low frequency relative to NHEJ and the requirement for the donor vector to have long homology arms [55,56,57]. Hence, NHEJ-mediated knock-in, which is more efficient than HDR, and simple donor vector structures have been used in various organisms [5,58,59,60]. In poultry, NHEJ-mediated knock-in was first reported in 2018. In that study, a green fluorescent protein (GFP) expression cassette was targeted to a locus between DNAJ homolog subfamily A member 1 (*DNAJA1*) and DNA replication regulator and spliceosomal factor (*SMU1*) of the Z chromosome, resulting in the successful production of GFP-expressing chickens that can be used as an avian sexing model [61].

**Figure 1:**
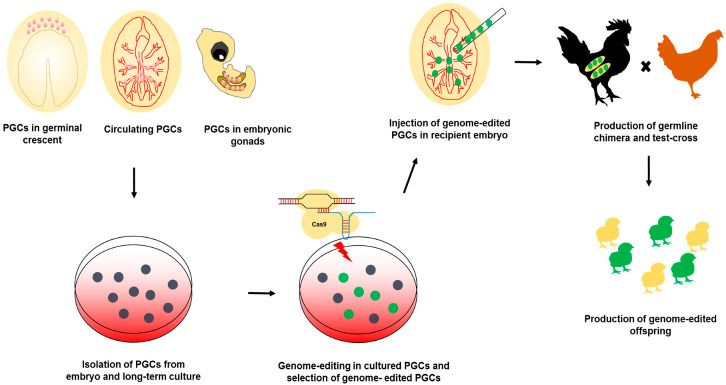
Schematic illustration of primordial germ cell (PGC)-mediated genome-editing in poultry. PGCs can be isolated from several stages of embryo and cultured in vitro. Genome-editing tools can be applied to cultured PGCs and genome-edited PGCs were enriched by using in vitro selection. The enriched genome-edited PGCs are injected into the bloodstream of recipient embryo and germline chimeras are produced. By mating with wild-type chicken, the genome-edited offspring finally produced.

Above all, these studies showed that CRISPR/Cas9-mediated genome editing, including targeted mutagenesis and HDR/NHEJ-mediated gene targeting, could be successfully conducted in cultured PGCs to produce genome-edited poultry (Figure 1).

### 3.2. Genome Editing in Poultry Using Other Methods

Although cultured PGCs are powerful tools for performing genome editing in poultry, there are some drawbacks to this approach. Among avian species, only chicken PGCs have been successfully cultured over the long term in vitro. In other poultry species, such as quail, PGCs cannot be cultured for many passages; consequently, it is difficult to select and amplify genome-edited PGCs. In addition, PGC-mediated methods are time-consuming, requiring selection of genome-edited PGCs, microinjection, and rearing of G0 germline chimeras to sexual maturity to obtain genome-edited offspring. Therefore, it is necessary to develop novel methods for the production of genome-edited poultry.

**Figure 2:**
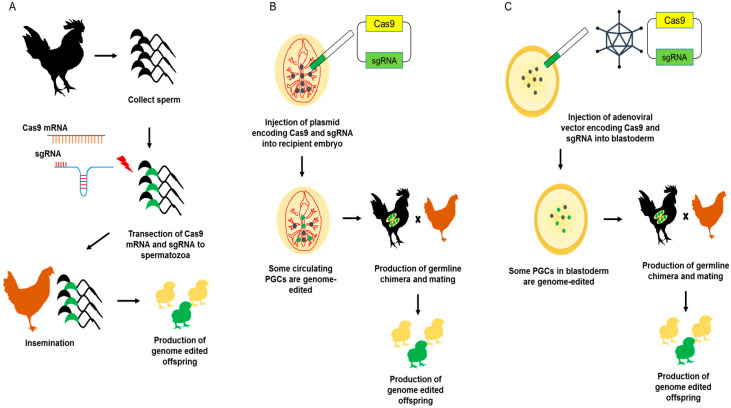
Schematic illustration of cultured-PGCs free genome-editing method in poultry. (**A**) Schematic illustration of STAGE. The spermatozoa collected from roosters are transfected with Cas9 mRNA and single guide RNA (sgRNA). The transfected spermatozoa were inseminated to adult hen and genome-edited offspring finally produced. (**B**) Schematic illustration of in vivo germ cell transfection. The plasmid encoding Cas9 and sgRNA is injected in recipient embryo bloodstream. Then, some circulating PGCs are transfected and genome-edited. After that, the germline chimera will be produced and genome-edited offspring finally produced after mating. (**C**) Schematic illustration of adenoviral vector injection. The adenoviruses containing vector encoding Cas9 and sgRNA are injected in the blastoderm and some PGCs in the blastoderm are infected with adenovirus and genome-edited. After that, the germline chimera will be produced and genome-edited offspring finally produced after mating.

One method for producing genome-edited birds is Sperm Transfection–Assisted Gene Editing (STAGE), which involves direct transfection of spermatozoa with Cas9 mRNA and sgRNA (Figure 2A). Using the transfected sperm for insemination, genome-edited progeny can be produced directly, a major advantage of STAGE relative to PGC-mediated genome editing. Using STAGE, genome editing was performed successfully in chicken embryos, but the efficiency of the production of genome-edited offspring was quite low, and additional improvement is still required [62].

Another method involves direct injection of plasmids into embryonic blood vessels (Figure 2B). Injection of Tol2 transposon and transposase plasmids into recipient embryos with lipofectamine can transform circulating PGCs and produce transgenic chickens [63]. A recent study reported that co-injection of a Tol2 transposon plasmid containing Cas9 and sgRNA expression cassettes and a transposase plasmid with lipofectamine can produce G1 progeny that stably express Cas9 and sgRNA. Using this method, both transgenic and non-transgenic genome-edited progenies are obtained in G2 [64].

Recently, adenovirus-mediated genome-editing methods have been developed (Figure 2C) [65]. The optimized adenoviral CRISPR/Cas9 vector, which expresses Cas9 and an sgRNA targeting the melanophilin (*MLPH*) gene, was injected into the blastoderm of the quail embryo. After hatching, 45% of chimeric quail (G0) produced genome-edited progeny, and germline transmission efficiency was up to 10%. Additionally, *MSTN* knockout quail, which have higher body weight and muscle mass, have been successfully produced using an adenoviral CRISPR/Cas9 vector system [66]. These results are important because the germline chimera efficiency is higher than those of other PGC-free methods, and the method has the potential to be broadly applied across poultry species. Additionally, adenovirus can be applied to postnatal gene editing in poultry [67]. Xu et al. adenoviral vector expressing *MSTN* gene-targeting sgRNA and SpCas9 was injected into the chick leg muscle, resulting in successful knockout of *MSTN* in that tissue.

To summarize, genome editing based on CRISPR/Cas9 technology has been successfully performed in poultry, resulting in a variety of genome-edited poultry for several purposes. As we discuss in the next section, these genome-edited poultry will be used in industries such as agriculture and biomedicine.

## 4. Application of Genome-Edited Poultry in Industries

In the future, the world population will continue to grow, and the demand for animal food products will increase accordingly. The FAO estimates that by 2050, the world population will be 9.7 billion, and the demand for animal food products will increase by 70% [1]. In preparation for this increased demand, economical traits such as productivity, disease resistance, and heat tolerance must be improved. Based on genome sequencing technology, researchers can find genetic variants that contribute to improved economical traits and use this DNA information for selective breeding. Genome editing can be harmonized with selective breeding because it can precisely edit target sites that have been identified by genome sequencing data and introduce novel alleles related to economically important traits without retention of transgenes. Several studies have used genome-editing technology in livestock to confer desirable traits such as disease resistance and heat tolerance, with the goal of improving productivity; in the future, this type of research will be greatly accelerated [4].

In poultry, genome editing has been performed to increase muscle productivity, feed conversion ratio, and disease resistance. *MSTN* encodes a negative regulator of muscle development, and knockout of *MSTN* significantly increases muscle mass in several animal species [68]. Hence, *MSTN* is a major target for improving the productivity of livestock [69,70,71,72]. In the case of poultry, knockout of *MSTN* and *G0S2* has been successfully performed. Muscle mass significantly increased in *MSTN* knockout chicken and quail, and fat composition was reduced in *G0S2*-knockout chicken [52,53,66]. These days, genome sequencing technologies have been rapidly developed and applied to poultry breeding to find genetic markers that influence productivity [73]. These genetic markers can be edited simultaneously using the CRISPR/Cas9 system. The combination of genomics and genome editing will further accelerate poultry breeding.

Viral diseases in poultry cause enormous economic loss and have the potential to significantly decrease poultry productivity. Genome editing can confer resistance to viral infection by modifying host factors that are crucial for viral entry or replication [74]. Viruses bind to host cell receptor molecules to gain entry into target cells [75]. Since the virus–receptor interaction is highly specific, deletion of the receptor can specifically prevent viral infection [76,77]. Thus, CRISPR/Cas9-mediated genome editing can be used to develop disease-resistant avian models by targeting host receptors. In preliminary studies in DF1 chicken fibroblasts, precise gene editing of the *chNHE1*, avian leucosis virus (ALV) subgroup receptor *tva*, *tvb*, and *tvc* genes using CRISPR-Cas9 conferred resistance to infection by ALV subgroup J (ALV-J), A (ALV-A), B (ALV-B), and C (ALV-C), respectively [78,79,80]. Based on these preliminary studies, genome-edited chickens were developed that harbored a precise deletion in tryptophan at residue 38 (W38) in chNHE1 and were resistant to ALV-J infection [7]. In the case of influenza A virus (IAV), host factor ANP32A plays a critical role in supporting the vPol activity of IAV [81,82,83]. In chicken ANP32A, an additional 33 amino acids are present between the leucine-rich repeats and C-terminal acidic region, and when these 33 amino acids are deleted, IAV replication in avian cells is significantly disrupted [81]. It has been speculated that if these 33 amino acids could be precisely deleted by genome editing, an IAV-resistant chicken could be produced [81,82,83]. These results imply that when host factors are identified as critical for viral entry or replication, we can successfully develop disease-resistant lines by genome editing. Additionally, when these host factors are edited simultaneously, it will be possible to develop poultry that are resistant to multiple diseases [84].

Recently, as awareness of animal welfare has increased, consumers have expressed a preference for purchasing products from livestock that were raised under beneficial conditions with high quality of life. In the case of laying hens, male chicks have historically been selected out and euthanized after hatching. This practice is now perceived as unethical, and research on the sex discrimination in the embryonic stage has been performed. Using genome editing, GFP expression cassettes have been precisely knocked into the Z chromosome in chicken. When GFP-expressing ZW females are mated with wild-type ZZ male, the male progeny express GFP and can be recognized by fluorescence-detecting devices at the embryonic stage [61].

On the other hand, an individual laying hen produces an average of 300 eggs per year, and the hens are easy to scale up due to their short generation time and small body size. Additionally, the relatively simple protein composition of egg white facilitates purification of the target protein. Since on these characteristics, the chicken egg has been recognized as an optimal bioreactor for production of therapeutic proteins [85,86]. Each egg white contains an average of 3.5 g of protein, of which ovalbumin constitutes half [85,87]. Due to the predominance of ovalbumin, researchers have attempted to express therapeutic proteins under the control of a synthetic ovalbumin promoter [8,9,10,11,12]. Recently, knock-in of a therapeutic protein at the ovalbumin locus, enabling expression of the protein under the control of the endogenous ovalbumin promoter, was successfully performed [14].

In addition to economical traits for therapeutic protein production, the *N*-glycosylation pattern of egg white proteins is also beneficial for certain therapeutics. In contrast to other vertebrates, chickens do not produce non-human glycans such as *N*-glycolylneuraminic acid (NGNA) and α-1,3 galactose (α-1,3-Gal), which can induce a significant immune response in human subjects [88,89]. Therefore, protein drugs derived from egg white are less likely to induce an adverse immune response in humans. Additionally, the *N*-glycosylation of egg white proteins has no core fucosylation [90]. This unique property can be exploited to produce therapeutic monoclonal antibodies that have antibody-dependent cell cytotoxicity (ADCC) as a main mode of actions, e.g., anticancer monoclonal antibodies [10]. Recently, taking advantage of egg white protein’s unique *N*-glycosylation pattern, the lysosomal acid lipase was produced and purified from egg white, and ultimately successfully commercialized [13]. If protein pharmaceuticals can be produced by a genome-edited chicken in which the major egg white protein locus has been targeted, it will be possible to produce protein pharmaceuticals with higher efficiency; moreover, these products will benefit from the egg white’s unique *N*-glycosylation pattern.

Additionally, because chickens and humans are phylogenetically distant from each other, it is easier to find an optimal antigen binding region for therapeutic antibodies against antigens that are highly conserved between human and mouse [91]. To produce humanized chicken for such applications, the variable region (V region) of the immunoglobulin heavy chain (IgH) was targeted, and a *loxP* site was inserted into that region using CRISPR/Cas9-mediated HDR [54]. These chickens will be used to discover optimal therapeutic antibody after insertion of human IgH V region by site-specific recombination.

## 5. Conclusions

CRISPR/Cas9-mediated genome-editing technology has developed rapidly over the past decade, and has been applied to the production of various genome-edited livestock, including poultry. Poultry is a major source of protein, and this role will become even more important in the future. Genome editing in poultry provides numerous opportunities to solve food shortage problems in agriculture. By combining advanced animal genomics based on genome sequencing technology, genome editing and animal breeding can be combined with each other to generate novel poultry lines with desirable traits such as heat tolerance or disease resistance. In addition, genome-edited poultry has potential as an alternative bioreactor platform for production of therapeutic proteins in eggs, as poultry bioreactors can overcome the limitations of mammalian cell culture systems related to *N*-glycosylation patterns and production costs. Development of valuable poultry bioreactors will become more active, and this new platform will soon be available for adoption by the pharmaceutical industry.

**Figure 3:**
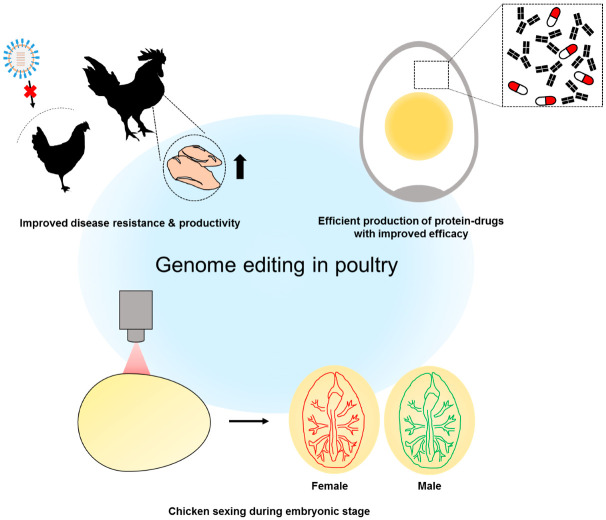
Schematic illustration for future application of genome-edited poultry to industries. Genome editing in poultry can improve disease resistance and meat productivity. By targeting egg white protein genes, genome edited poultry can economically produce protein drugs with improved biological efficacy. When the reported genes are targeted to the Z chromosome, the male embryo can be screened out before hatching by detecting fluorescence during incubation.

Collectively, rapidly developing genome-editing technology will accelerate progress in the poultry biotechnology field as well, opening up new opportunities for poultry to contribute to various industries (Figure 3).
